# A semi-virtual two dimensional gel electrophoresis: IF–ESI LC-MS/MS

**DOI:** 10.1016/j.mex.2017.08.004

**Published:** 2017-08-31

**Authors:** Stanislav Naryzhny, Victor Zgoda, Artur Kopylov, Elena Petrenko, Аlexander Archakov

**Affiliations:** a Orekhovich Institute of Biomedical Chemistry of Russian Academy of Medical Sciences, Pogodinskaya 10, Moscow, 119121, Russia; b B.P. Konstantinov Petersburg Nuclear Physics Institute, National Research Center “Kurchatov Institute”, Orlova roscha, Gatchina, Leningrad region, 188300, Russia

**Keywords:** A semi-virtual two dimensional gel electrophoresis: IF–ESI LC-MS/MS, Proteoforms, Large-scale, Identification, IEF, Shotgun, 2DE

## Abstract

A method for increasing the productivity of ESI LC-MS/MS (electrospray ionization-liquid chromatography-tandem mass spectrometry) was proposed and applied.

After IF (isoelectric focusing) of the sample using IPG (immobilized pH gradient) strip, the strip was cut to sections, and every section was treated according to trypsinolysis protocol for MS/MS analysis. The peptides produced were further analyzed by ESI LC-MS/MS. The procedure allows to:

• identify many more proteins and proteoforms compared to shotgun analysis of extracts.
• build a semi-virtual 2DE map of identified proteins.

## Graphical abstract

**Figure:**
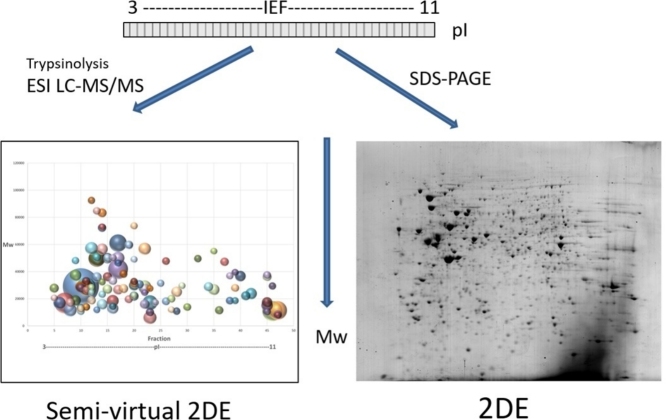

## Method details

High resolution separation of complex protein samples has allowed the 2DE technique to be a leading approach in the study of proteomics since its introduction in 1975 by O’Farrell [1]. Separation is a main function of 2DE and a first step towards obtaining the necessary information about proteins. Together with large scale identification methods, like mass spectrometry, 2DE was a work horse of proteomics for a long time [2], [3]. As high tech mass spectrometry can execute some 2DE functions, it is possible to perform 2D analysis in non-classical way. One example is so called “Virtual 2-D Gel Electrophoresis” [4], [5], where isoelectrofocusing in IPG strips is used as a first dimension, but the conventional second separation by SDS-PAGE is replaced by MALDI-MS (matrix assisted laser desorption ionization- mass spectrometry). Usage of ESI LC-MS/MS, instead of MALDI-MS, in combination with classical electrophoretic methods allows the revelation of more information; especially when very complex samples like mammalian cell extracts are analyzed [6], [7], [8], [9]. In particular, in comparison to the classical shotgun analysis semi-virtual 2DE allows the acquisition of not just protein profiles but also proteoform profiles.

## Chemicals and materials

All reagents used were obtained from “Sigma-Aldrich” (St. Louis, MO, USA), unless another manufacturer is specified. The remaining reagents were obtained from the following companies: “Thermo Scientific Pierce”, (Rockford, IL, USA): dithiothreitol (DTT), protease inhibitor cocktail; “GE Healthcare ‘(Chicago, IL, USA): IPG DryStrip (gel strips), IPG-buffers (ampholytes), DryStrip-coating liquid; “Promega Corp’ (Madison, WI, USA): Trypsin Gold.

## Procedure

**Fig. 1:**
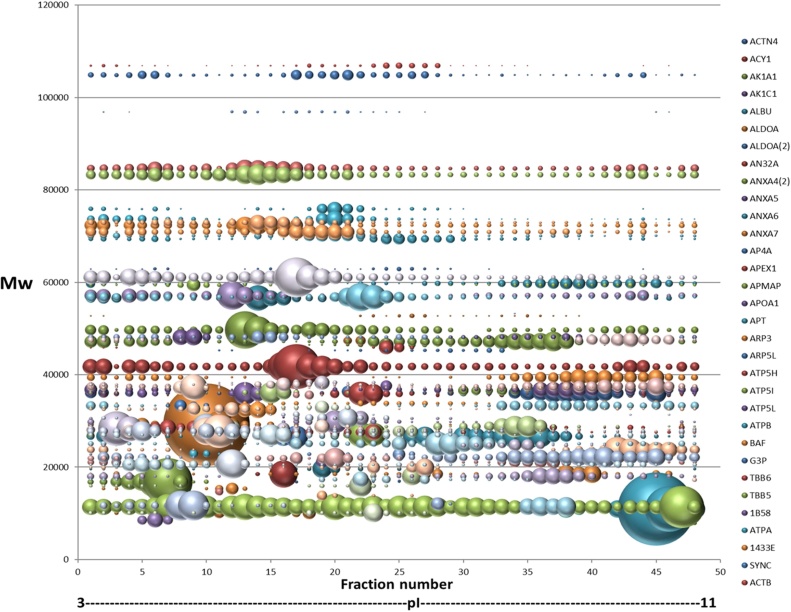
2DE virtual representation of HepG2 most abundant proteoforms after IF and following ESI LC-MS/MS. A ball size is proportional to the proteoform abundance (emPAI).

1. Human hepatocellular carcinoma (HepG2) cells were cultured in medium DMEM/F12 supplemented with 10% fetal bovine serum (FBS) and 100 U/ml penicillin) under standard conditions (5% CO_2_, 37 °C). To prepare cell samples for protein extraction, the cells were detached with 0.25% Trypsin-EDTA solution, and washed 3 times with PBS.
2. 10 million cells containing approximately 2 mg of protein, were treated with 100 μL of lysis buffer (7 M urea, 2 M thiourea, 4% CHAPS, 1% DTT, 2% ampholytes, pH 3–10, protease inhibitor mixture).
3. 50 μL of the cell lysate (1000 μg of protein) was mixed with rehydrating buffer (7 M urea, 2 M thiourea, 2% CHAPS, 0.3% DTT, 0.5% IPG buffer, pH 3–11 NL, 0.001% bromophenol blue) in a final volume of 400 μL.
4. An IPG strip (Immobiline DryStrip 3–11 NL, 24 cm, ‘GE Healthcare’) was placed onto the sample, gel side down, and was passively rehydrated for 5 h at 4 °C
5. Proteins were separated by IEF using IPGphor (GE Healthcare), which was programmed as follows: first step – 500 V, 8 h, second step – gradient to 1000 V, 1 h, third step – gradient to 10000 V, 3 h, fourth step – 10000 V, 4 h, temperature 20 °C, thereafter maintained at the voltage 500 V
6. After IEF, the strip was cut into 48 equal sections using scissors, and each section was transferred to Eppendorf tube.
7. For complete reduction, 300 μL of 3 mM DTT, 100 mM ammonium bicarbonate was added to each sample.
8. Incubated at 50 °C for 15 min.
9. For alkylation 20 μL of 100 mM iodoacetamide (IAM) were added to the same tube.
10. Incubated in the dark at room temperature for 15 min.
11. Liquid was removed and replaced with 300 μL of 50% acetonitrile, 50 mM ammonium bicarbonate.
12. Incubated for 15 min.
13. Liquid was removed and replaced with 150 μL of 100% acetonitrile.
14. Incubated for 10 min or until all gel slices are white.
15. Liquid was removed and gel was desiccated in Speed Vac (Thermo Scientific) for 5 min.
16. For digestion, 0.1 mg/mL stock trypsin was diluted 1:10 into 25 mM ammonium bicarbonate.
17. 100 μL of diluted trypsin was added into each tube.
18. Samples were incubated for 4–24 h at 37 °C (overnight).
19. Supernatants were collected to new labeled 0.5 mL tubes. Supernatants may contain peptides that have diffused out of the gel slices.
20. Peptides were extracted by adding 150 μL of 60% acetonitrile, 0.1% trifluoroacetic acid (TFA) to each tube containing gel slices.
21. Incubated for 15 min.
22. Extracts were removed and combined with the supernatants in the new labeled tubes.
23. Extraction was repeated.
24. Extracts were dried in Speed Vac.
25. For samples analyzed by LC/MS, the peptides were reconstituted in 20 μL of 0.1% TFA.
26. Tryptic peptides were separated using reversed phase nano-LC gradients and analyzed online by Orbitrap Q-Exactive Plus mass spectrometer.
27. Protein identification and relative quantification were performed using Mascot “2.4.1” (Matrix Science) and emPAI.
28. A table with information about all detected protein proteoforms was built (Table 1). All proteins detected in the same section were given the pI of this section. Accordingly, the same proteins detected in different sections were considered as different proteoforms.
29. Based on information from NeXtProt about Mw of detected proteins, a semi-virtual 2DE map was constructed. An example of such a map for major proteins expressed in HepG2 cells is shown in Fig. 1.

## Method validation

The method was validated in our laboratory using several cell lines. After protein identification, a list of 32,652 proteoforms (products of 3962 genes) was generated from HepG2 cells. Only 1242 proteins were identified in parallel shotgun experiment.

## Additional information

For convenience, the IPG strip (3-11 NL, 24 cm) was only cut into 48 sections, as ESI LC-MS/MS itself is a time consuming process. The range of approximately 0.166 pH units per section was obtained in this case. This range can be narrowed using a bigger number of sections. It will give a better resolution allowing more proteoforms to be detected, as the difference in pIs between proteoforms of the same protein can be less than 0.05 pH units [10], [11].

An alkylation step is optional, but it is highly recommended for better identification of proteins. It improves the recovery of cystine-containing peptides and minimizes the appearance of unknown masses from disulfide bond formation and side chain modifications.
